# New chromium(III)-based catalysts for ethylene oligomerization

**DOI:** 10.1038/s41598-020-73420-6

**Published:** 2020-10-06

**Authors:** Jacek Malinowski, Dagmara Jacewicz, Barbara Gawdzik, Joanna Drzeżdżon

**Affiliations:** 1 Faculty of Chemistry, University of Gdańsk, Wita Stwosza 63, 80-308 Gdańsk, Poland; 2 Institute of Chemistry, Jan Kochanowski University, Świętokrzyska 15 G, 25-406 Kielce, Poland

**Keywords:** Analytical chemistry, Catalysis, Coordination chemistry

## Abstract

The report focuses on the new precatalysts for ethylene oligomerization. The five chromium(III) complex compounds containing the following ligands: dipicolinate anion, oxalate anion, 5-aminopyridine-2-carboxylate anion, 2,2′-bipyridine and 4,4′-dimethoxy-2,2′-bipyridine have been examined towards catalytic activity for ethylene oligomerization. The chromium(III) complexes have been activated by modified methylaluminoxane. The obtained oligomers have been investigated by MALDI-TOF–MS, thermal analysis and infrared spectroscopy. The results revealed that the examined chromium(III) complexes are highly active catalysts for ethylene oligomerization. The values of catalytic activities of the examined complexes are in the range 1860 – 3798 g∙mmol^-1^∙h^-1^∙bar^-1^.

## Introduction

Preparation of polyethylene oligomers is of great significance in industry^1^. Selective production of linear α-olefins is particularly important^2–4^. Linear α-olefins are used in the industry for the production of surfactants, lubricants, adhesives and plasticizers^5^.

Non-metallocene chromium(III) complexes used as catalysts in olefin polymerization are new generation catalysts^6^. The chromium(III) complex with triptycenyl and 2-pyridylmethyl has the highest catalytic activity which equal to 6970 g∙mmol^−1^∙h^−1^∙bar^−1^^6^. Other complexes have relatively low values of catalytic activities. Cr[N(SiMe_3_)_2_]_2_I_2_ exhibits 43 g∙mmol^−1^∙h^−1^∙bar^−1^ activity, chromium(III) complex with 2-(1-isopropyl-2-benzimidazolyl)-6-(1-(arylimino)ethyl)pyridines has the activity equal to 114 g∙mmol^−1^∙h^−1^∙bar^−1^, bis(salicylaldiminato) chromium(III) complex 96 g∙mmol^−1^∙h^−1^∙bar^−1^ and bis(phosphino)amide complex of chromium(III) has the 500 g∙mmol^−1^∙h^−1^∙bar^−1^ activity^7–11^. The polyethylene obtained during the reaction undergoing at 50 °C and catalysed by bis(phosphino)amide complex of chromium(III) is the low-molecular-weight polyethylene^12,13^.

In 2019 Arrozi together with colleagues, they developed new ethylene oligomerization catalysts that occur under mild conditions. The role of catalysts was played by porous or non-porous coordination polymers / metal – organic frameworks based on nickel^14^. The intrinsic activity of these catalysts was in the range of 1–182 per mmol Ni per h. In 2020 new catalysts cointaing Ni i.e. non-ordered mesoporous Ni-AlSiO_2_, were prepared and used in the ethylene oligomerization process at moderate temperature and pressure (at 250 °C and 15 bar)^15^.

During our earlier research we have found that complex compounds such as [Cr(dipic)_2_][Cr(bipy)(dipic)(H_2_O)] ∙ 2 H_2_O, [Cr(dipic)_2_]Hdmbipy ∙ 2.5 H_2_O, *cis*-K[Cr(C_2_O_4_)(OH_2_)_2_] and [Cr(dipic)(H_2_O)_3_]Cl ∙ 2 H_2_O (dipic denotes dipicolinate anion, bipy = 2,2′-bipyridine, Hdmbipy = 4,4′-dimethoxy-2,2′-bipyridine with an additional proton) exhibit catalytic activity in the oligomerization reaction of 2-chloro-2-propen-1-ol^16–19^. These precatalysts are activated by MMAO-12 (modified methylaluminoxane). The reaction undergoes at atmospheric pressure and at room temperature.

This report is a continuation of our earlier research on a group of chromium(III) complex compounds. In this article we describe the results of the studies on the catalytic activity of the following complexes: [Cr(dipic)_2_][Cr(bipy)(dipic)(H_2_O)] ∙ 2 H_2_O, [Cr(dipic)_2_]Hdmbipy ∙ 2.5 H_2_O, *cis*-K[Cr(C_2_O_4_)(OH_2_)_2_], [Cr(dipic)(H_2_O)_3_]Cl ∙ 2 H_2_O and (5-aminopyridine-2-carboxylate)_3_ chromium(III). The catalytic activity of these compounds has been examined and determined in the ethylene oligomerization. The obtained products have been characterized by several methods i.e. MALDI-TOF–MS, thermal analysis and infrared spectroscopy.

## Results and discussion

**Figure 1:**
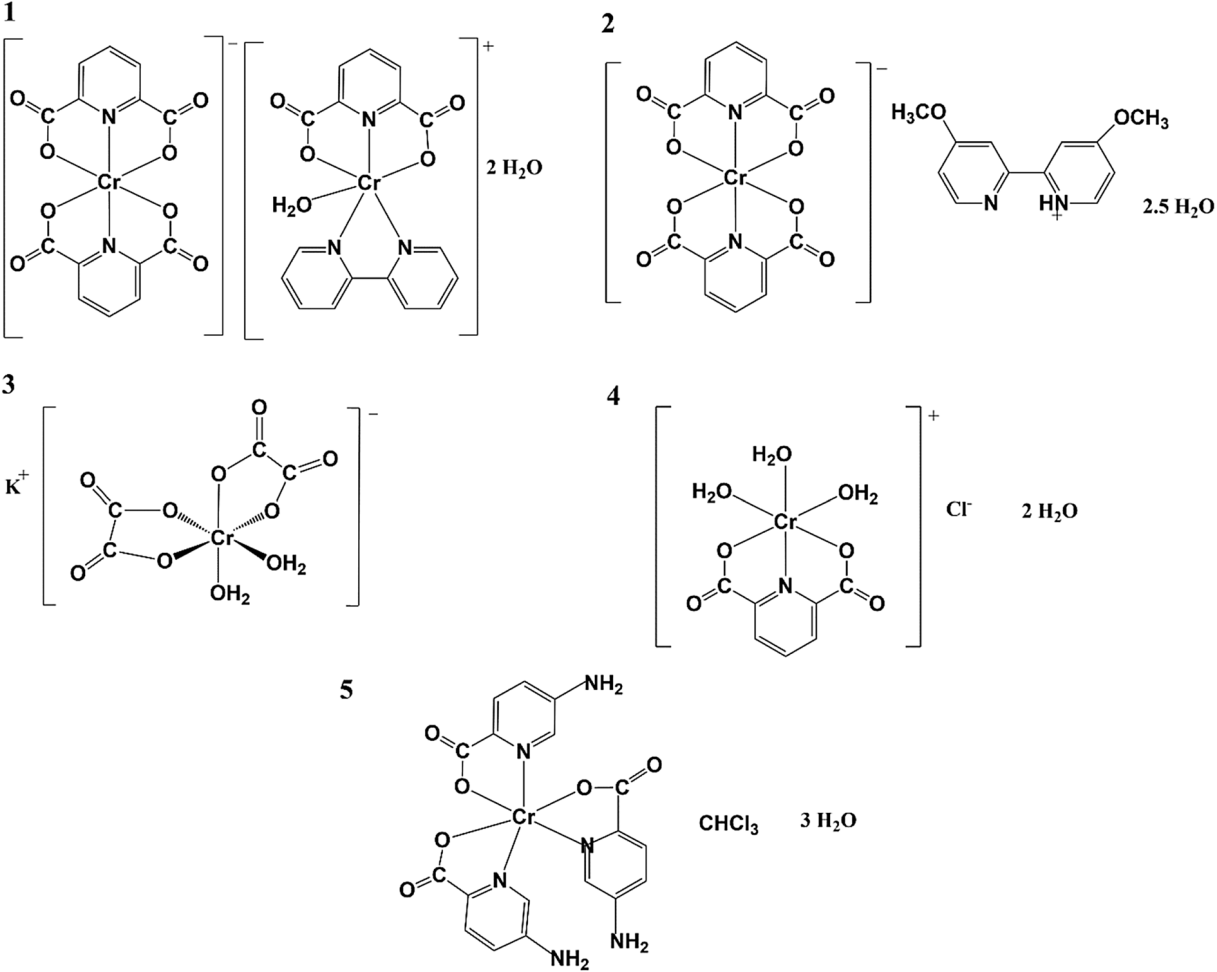
The structures of complex compounds acting as catalysts in ethylene oligomerization: (1) [Cr(dipic)_2_][Cr(bipy)(dipic)(H_2_O)] ∙ 2 H_2_O, (2) [Cr(dipic)_2_]Hdmbipy ∙ 2.5 H_2_O, (3) *cis*-K[Cr(C_2_O_4_)(OH_2_)_2_], (4) Cr(dipic)(H_2_O)_3_]Cl ∙ 2 H_2_O and (5) (5-aminopyridine-2-carboxylate)_3_ chromium(III).

The catalytic activity of the ethylene oligomerization reaction using chromium(III) complex compounds (Fig. 1) used as catalysts for the conducted reaction was calculated (Table 1) from the following mathematical relationship:

$$\text{catalytic activity}= \frac{{m}_{p}}{{n}_{M}\cdot t \cdot p}$$

where m_p_ – weight of oligomer sample [g], n_M_ – amount of millimoles of metal ions used in the oligomerization process [mmol], t – oligomerization process time [h], p – ethylene pressure applied during the reaction [bar].

**Table 1:** Data for the ethylene oligomerization catalyzed by chromium(III)-based complexes.

Compound	Oligomerization process time (h)	Catalytic activity (g∙mmol^-1^∙h^-1^ ∙bar^-1^)	Gas pressure (bar)
[Cr(dipic)_2_][Cr(bipy)(dipic)(H_2_O)] ∙ 2 H_2_O	1/3	2459	0.3
[Cr(dipic)_2_]Hdmbipy ∙ 2.5 H_2_O	1/3	3798	0.2
*cis*-K[Cr(C_2_O_4_)(OH_2_)_2_]	1/6	1860	0.2
[Cr(dipic)(H_2_O)_3_]Cl ∙ 2 H_2_O	1/6	2064	0.2
3 (C_18_H_15_CrN_6_O_6_) ∙ CHCl_3 _∙ 5 H_2_O	1/6	2703	0.2

Ethylene oligomers have been characterized by MALDI-TOF–MS. In the case of the ethylene oligomerization product catalyzed by [Cr(dipic)_2_][Cr(bipy)(dipic)(H_2_O)] ∙ 2 H_2_O during the analysis, DHB matrix—2,5-dihydroxybenzoic acid was used. In the registered mass spectrum we see a peak at m/z = 177.013, which comes from the matrix used during the analysis. Peaks at m/z = 273.022, m/z = 354.974, m/z = 449.338 contain 10, 13 and 16 mers respectively in the oligomer chain. The molecular peak at a m/z = 332.984 contains 11 mers in the oligomer chain. The main product contains 24 carbon atoms in the oligomeric chain. The molecular peak has a signal intensity of 9955 [a.u.].

**Figure 2:**
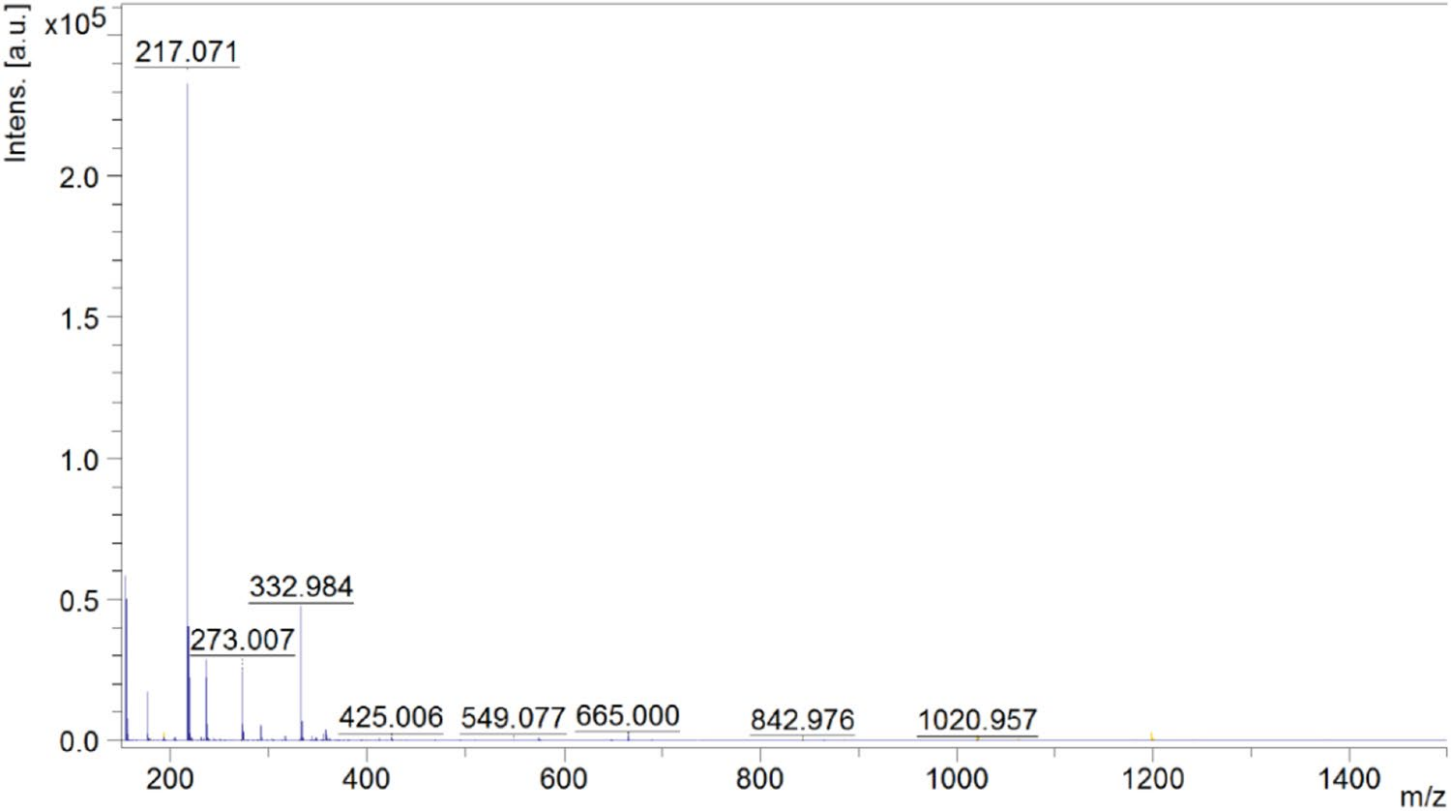
Mass spectrum of the ethylene polymerization product catalysed by [Cr(dipic)_2_]Hdmbipy ∙ 2.5 H_2_O.

The mass spectrum in Fig. 2 presents mass analysis of the ethylene oligomerization reaction using a catalyst [Cr(dipic)_2_]Hdmbipy ∙ 2.5 H_2_O. Mass analysis was carried out using the MALDI-TOF MS method. The DHB matrix, i.e. 2,5-dihydroxybenzoic acid was used during the tests. A peak at m/z = 273.007 is a signal from the matrix. The molecular peak, which occurs at = 217.071 m/z, contains 7 mers in the oligomer chain. The resulting ethylene oligomer contains 15 carbon atoms. The signal from the molecular peak was characterized by an intensity of 237,320 [a.u.]. The peak at m/z = 332.984 contained 12 mers in the chain, while the peak at m/z = 425.006 contained 15 mers in the oligomer chain.

The mass analysis of ethylene oligomerization using K[Cr(C_2_O_4_)(OH_2_)_2_] as a catalyst was performed on a CAA matrix. Signals at m/z = 379.066 and m/z = 304.266 came from the used matrix. The molecular peak occurs at m/z = 294.049 and it confirmed 10 mers in the oligomer chain and a signal intensity of 19,494 [a.u.]. This oligomer is built from 11 carbon atoms. The remaining peaks with m/z = 440.992, m/z = 524.111 and m/z = 643.994 contained 16 mers, 18 mers and 23 mers in their oligomer chain, respectively.

The mass spectrum of the ethylene polymerization product catalysed by [Cr(dipic)(H_2_O)_3_]Cl ∙ 2 H_2_O has been regisered on the DHB matrix. The molecular peak occurs m/z = 332.985 and it confirmed the presence of 11 mers in the oligomer chain which has 24 carbon atoms. A signal intensity is equal to 20,312 [a.u.]. Peaks at m/z = 273.009, m/z = 400.345 and m/z = 664.997 contained 10, 14 and 23 mers in the oligomer chain, respectively.

The CAA matrix was used in the case of the analysis of the ethylene oligomer obtained with (5-aminopyridine-2-carboxylate)_3_ chromium(III) complex. Peaks at m/z = 379.069 and 839.326 are signals from the used matrix. The molecular peak was characterized by 260.936 m/z. It can be concluded, that it contained 9 mers in the oligomer chain which is built from 18 carbon atoms. Its signal intensity was 27,146 [a.u.]. The peak at m/z = 294.054 contained 10 mers in the chain.

The oligomers obtained during these studies were tested by thermal analysis in order to determine the temperature gradient distribution characteristics of the tested samples. In the case [Cr(dipic)2][Cr(bipy)(dipic)(H_2_O)] ∙ 2 H_2_O (sample 1) used as the catalyst for the oligomerization reaction of ethylene, 31.48% of thermal decomposition up to 230.3 °C the oligomer tested. In the case of sample, where the compound [Cr(dipic)_2_]Hdmbipy ∙ 2.5 H_2_O (sample 2) was used as a catalyst in the temperature range up to 300 °C, 31.25% of the initial oligomer sample mass was decomposed. Sample where *cis*-K[Cr(C_2_O_4_)(OH_2_)_2_] (sample 3) was a catalyst for the reaction, mass lost was 36.9% as a result of thermal decomposition in the temperature range up to 330 degrees Celsius. In the case of samples where [Cr(dipic)(H_2_O)_3_]Cl ∙ 2 H_2_O (sample 4) and 3(C_18_H_15_CrN_6_O_6_) ∙ CHCl_3_ ∙ 5 H_2_O (sample 5) were used as catalysts. The product obtained by [Cr(dipic)(H_2_O)_3_]Cl ∙ 2 H_2_O decomposed thermally in 30.21% to 420 °C, while in the case of sample of oligomer obtained by 3(C_18_H_15_CrN_6_O_6_) ∙ CHCl_3_ ∙ 5 H_2_O mass reduced by 40.49% in the range of 0 °C to 400 °C. Sample 1, in the third stage of thermal decomposition, underwent mass loss of 20.31% in the temperature range 280 °C – 720 °C , that in the last stage up to 990 °C, the sample decomposes by 4.52%. Sample 2 also decomposed in 4 stages. The third stage is a weight loss of 19.31% in the temperature range 280 °C—640 °C, and then slightly decomposing the sample to 3.01% weight loss—decomposition carried out to 860 °C. In the case of sample 3, thermal decomposition took place in three stages. Stage 2 is a weight loss of 9.52% in the temperature range of 320 °C—680 °C. The last stage is sample distribution in 3.63% to 910 °C. Sample 4 was broken down in three stages. The third stage was 6.34% weight loss in the temperature range from 400 °C to 955 °C. The fifth sample in the temperature range 380 °C -520 °C decomposed by 4.54%, so that in the last stage to 905 °C the sample mass decreased by 6.80%. A different number of stages of thermal decomposition of the studied products of the ethylene oligomerization reaction may result from different branching and structure of oligomeric chains. Oligomers formed as a result of the ethylene oligomerization reaction catalysed by complex compounds were thermally decomposed with the probable separation of the following products: carbon monoxide(IV), carbon monoxide(II) and water.

The ethylene oligomers have been studied by IR. The obtained results confirm the composition of the oligomer chain (Tables 2, 3, 4, 5, and 6).

**Table 2:** IR results for the ethylene oligomer obtained using [Cr(dipic)_2_][Cr(bipy)(dipic)(H_2_O)] ∙ 2 H_2_O as a catalyst.

The wavenumber (cm^−1^)	The vibrations
2929.38	Strong asymmetric CH_2_ stretching vibrations in the oligomer chain
2858.31	Strong asymmetric CH_2_ stretching vibrations in the oligomer chain
1466.23	Strong CH_2_ deformations in the oligomer chain
816.39	CH_2_ rocking vibrations in the oligomer chain
859.17	CH_2_ rocking vibrations in the oligomer chain

**Table 3:** IR results for the ethylene oligomer obtained using [Cr(dipic)_2_]Hdmbipy ∙ 2.5 H_2_O as a catalyst.

The wavenumber (cm^−1^)	The vibrations
2925.78	Strong asymmetric CH_2_ stretching vibrations in the oligomer chain
2853.37	Strong asymmetric CH_2_ stretching vibrations in the oligomer chain
1467.68	Strong CH_2_ deformations in the oligomer chain
1379.19	Weak symmetric terminal CH_3_ deformation in the oligomer chain
721.66	CH_2_ rocking vibrations in the oligomer chain

**Table 4:** IR results for the ethylene oligomer obtained using cis-K[Cr(C_2_O_4_)(OH_2_)_2_] as a catalyst.

The wavenumber (cm^−1^)	The vibrations
1514.59	Strong CH_2_ deformations in the oligomer chain
1426.87	Strong CH_2_ deformations in the oligomer chain
857.02	CH_2_ rocking vibrations in the oligomer chain

**Table 5:** IR results for the ethylene oligomer obtained using [Cr(dipic)(H_2_O)_3_]Cl ∙ 2 H_2_O as a catalyst.

The wavenumber (cm^−1^)	The vibrations
2950.06	Strong asymmetric CH_2_ stretching vibrations in the oligomer chain
2850.03	Strong asymmetric CH_2_ stretching vibrations in the oligomer chain
1496.29	Strong CH_2_ deformations in the oligomer chain
1469.54	Weak symmetric terminal CH_3_ deformation in the oligomer chain
696.19	CH_2_ rocking vibrations in the oligomer chain

**Table 6:** IR results for the ethylene oligomer obtained using 3 (C_18_H_15_CrN_6_O_6_) ∙ CHCl_3 _∙ 5 H_2_O as a catalyst.

The wavenumber (cm^−1^)	The vibrations
1517.72	Strong CH_2_ deformations in the oligomer chain
1424.66	Weak symmetric terminal CH_3_ deformation in the oligomer chain
858.18	CH_2_ rocking vibrations in the oligomer chain

## Conclusions

All chromium(III) complexes studied in this report after activation by MMAO-12 are highly active catalysts for ethylene oligomerization. The most active catalyst is [Cr(dipic)_2_]Hdmbipy ∙ 2.5 H_2_O which has the catalytic activity equal 3798* g*∙mmol^-1^∙h^-1^ ∙bar^-1^. The oligomerization processes catalysed by chromium(III) complexes described in this article undergo fast i.e. from 15 to 20 min. The polyethylene oligomers obtained consist of 8 to 12 mers. The 12-mer chains are obtained using [Cr(dipic)_2_][Cr(bipy)(dipic)(H_2_O)] ∙ 2 H_2_O and [Cr(dipic)(H_2_O)_3_]Cl ∙ 2 H_2_O as catalysts. The oligomers decompose thermally in four stages, except for samples 3 and 4 which decompose thermally in 3 stages. IR analysis of oligomers confirmed the composition of the chain.

## Methods

### Syntheses

The complexes: [Cr(dipic)_2_][Cr(bipy)(dipic)(H_2_O)] ∙ 2 H_2_O, [Cr(dipic)_2_]Hdmbipy ∙ 2.5 H_2_O, *cis*-K[Cr(C_2_O_4_)(OH_2_)_2_] and [Cr(dipic)(H_2_O)_3_]Cl ∙ 2 H_2_O have been synthesized according to the procedures described in the literature^16–19^.

The (5-aminopyridine-2-carboxylate)_3_ chromium(III) (3 (C_18_H_15_CrN_6_O_6_) ∙ CHCl_3 _∙ 5 H_2_O) complex has been synthesized as follows. 5-Aminopyridine-2-carboxylic acid (4 mmol, 0.55 g) was dissolved in ethanol and added to the aqueous solution containing CrCl_3_ ∙ 6 H_2_O (2 mmol, 0.54 g). All mixed up. Then, the mixture was heated at 50° C and mixed for 15 min. In the next step, the mixture was cooled and trichloromethane and methanol in the volume ratio 1 : 2 was added. After 2 months the (5-aminopyridine-2-carboxylate)_3_ chromium(III) complex precipitated.

The synthesized chromium(III) complexes were examined by elemental analysis (the Vario EL analyzer Cube (CHNS)). The elemental analysis results are follows: [Cr(dipic)_2_][Cr(bipy)(dipic)(H_2_O)] ∙ 2 H_2_O : C, 45.88%, H, 2.75%, N, 8.42%. Anal. Calcd.: C, 45.95%, H, 2.84%, N, 8.65%.[Cr(dipic)_2_]Hdmbipy ∙ 2.5 H_2_O : C, 48.25%, H, 3.56%, N, 8.57%. Anal. Calcd.: C, 48.41%, H, 3.72%, N, 8.69%. *Cis*-K[Cr(C_2_O_4_)(OH_2_)_2_] : C, 15.19%, H, 1.80%. Anal. Calcd.: C, 15.91%, H, 1.95%.[Cr(dipic)(H_2_O)_3_]Cl ∙ 2 H_2_O : C, 26.64%, H, 3.82%, N, 4.31%. Anal. Calcd.: C, 24.52%, H, 3.79%, N, 4.09%. 3 (C_18_H_15_CrN_6_O_6_) ∙ CHCl_3 _∙ 5 H_2_O : C, 42.81%, H, 46% N, 15.67%. Anal. Calcd.: C, 41.30%, H, 3.53%, N, 15.75%. Detailed crystallographic data on the structures of complex compounds used as catalysts in the ethylene oligomerization reaction are collected in the following references^16–18,20^.

## The oligomerization process

A solution of the complex as a catalyst was prepared by dissolving 1 μmol of the complex in 1 mL of toluene in a glass tube located above the magnetic stirrer. The entire polymerization process was carried out at atmospheric pressure, at room temperature and nitrogen atmosphere. In the next reaction stage, 1 mL of MMAO-12 solution was introduced. MMAO-12 was purchased from Sigma-Aldrich. It was 7 wt% aluminum in toluene.[(CH_3_)_0.95_(n-C_8_)H_17_]_0.05_AlO]_n_ is linear formula of MMAO-12. The molar ratio of [Al]/[Cr] was equal to 1000:1. The solution was allowed to mix for a while, then ethylene was added all the time until the process was complete. The reaction was terminated according to the procedure described in the literature^21^. The pressure ethylene was released and the catalytic system was quenched with water.

### MALDI-TOF–MS

MALDI-TOF–MS analysis was performed for ethylene oligomerization reaction products using chromium complexes as reaction catalysts. Sample analysis was performed on DHB (2,5-dihydroxybenzoic acid) and CAA (α-cyano-4-hydroxycinnamic acid) matrices^22^.

### Thermal analysis

Thermal analysis (TG) was performed on NETZSCH TG 209 instrument. Measurements were made in the temperature range from 0 °C to 1000 °C. The mass of samples subjected to thermal analysis was about 5 mg. Thermal analysis was carried out in an argon atmosphere.

### Infrared spectroscopy

IR analysis was carried out using the KBr pastil. Measurements were carried out from 4000 cm^-1^ to 600 cm^-1^.
